# Homology-directed repair in mouse cells increased by CasRx-mediated knockdown or co-expressing Kaposi’s sarcoma-associated herpesvirus ORF52

**DOI:** 10.1042/BSR20191914

**Published:** 2019-10-15

**Authors:** Hong Pan, Weina Yu, Ming Zhang

**Affiliations:** 1 State Key Laboratory for Conservation and Utilization of Subtropical Agro-bioresources, College of Life Science and Technology, Guangxi University, 100 Daxue Road, Nanning 530004, Guangxi, China; 2 Department of Assisted Reproduction, Shanghai Ninth People’s Hospital, Shanghai Jiao Tong University School of Medicine, Shanghai 200011, China; 3 College of Animal Science and Technology, Guangxi University, 100 Daxue Road, Nanning 530004, Guangxi, China

**Keywords:** CasRX, CRISPR/Cas9, Homology-directed repair, ORF52

## Abstract

Precise genome editing with directed base insertion or targeted point mutations can be achieved by CRISPR/Cas9-mediated homology-directed repair (HDR) and is of great significance in clinical disease therapy. However, HDR efficiency, compared with non-homologous end-joining (NHEJ), is inherently low. To enhance HDR, enabling the insertion of precise genetic modifications, we compared two strategies during surrogate reporter assays in mouse N2A cells: the suppression of DNA ligase IV, a key molecule in NHEJ, using the CasRx (*Ruminococcus flavefaciens* Cas13d) system, and co-expression of Kaposi’s sarcoma-associated herpesvirus (KSHV) ORF52 proteins. We found that suppression of DNA ligase IV promotes HDR efficiency by 1.4-fold. When co-expressed with the Cas9 system, ORF52 improved HDR efficiency by up to 2.1-fold. In addition, we used ORF52 co-expression to modify the *ACTB* and *Tubb3* genes of mouse N2A and E14 cells, which further increased HDR efficiency by approximately two- to four-fold. In conclusion, our data suggest that ORF52 co-expression is effective for enhancing CRISPR/Cas9-mediated HDR, which may be useful for future studies involving precise genome editing.

## Introduction

Therapeutic applications of CRISPR-Cas9 generally require the use of donor templates for precise mutation. Although programmable nucleases such as CRISPR-Cas9 can produce DNA double-strand breaks (DSBs), their repair is more likely to involve error-prone non-homologous end-joining (NHEJ) rather than homologous recombination (HR). Furthermore, the use of a DNA template donor combined with NHEJ often leads to the formation of insertions or deletions (indels) [1]. In clinical disease therapy, the insertion of precise genetic modifications by CRISPR-Cas9 is limited by the relatively low efficiency of homology-directed repair (HDR), and several investigations have been made to enhance HDR-mediated gene editing of CRISPR-Cas9 induced DSBs from donor templates. In some studies, cells have been synchronized prior to CRISPR-Cas9 delivery because HDR is active only during the late S/G_2_ phase of the cell cycle, whereas NHEJ activity occurs throughout the cell cycle, or the Cas9 variant expression has been limited to the S/G_2_ phase [2–4]. HDR occurs concurrently, not sequentially, with NHEJ and occurs more frequently in NHEJ-deficient cells [5]. Other studies have achieved higher rates of HDR by designing DNA repair templates with optimal homology arm lengths [6,7], or by chemically modulating the NHEJ and HDR pathways [8–11]. There are two types of NHEJ pathways: ligase IV-dependent NHEJ (C-NHEJ), which mediates DNA repair after DSBs in most cases, and the alternative NHEJ pathway (A-NHEJ), which requires DNA ligase I/III and is defined as any Ku- or Lig4-independent end-joining process [12]. Thus, DNA ligase IV, a key enzyme for C-NHEJ, was considered a potential target to improve HDR efficiency. However, Lig4 knockout cells showed a marked sensitivity to X-rays, bleomycin, VP-16 and ablated V(D)J recombination that is a physiologic process of DNA breakage and rejoining in which double-strand DNA breaks are formed as an intermediate [13,14]. Lig4 knockout mice are embryonic lethal, which has been associated with massive neuronal cell death, preventing the use of zygotes genetically deficient in DNA ligase IV as a strategy for making mutant mice [15]. Therefore, transient targeting of DNA ligase IV is an especially important strategy. Indeed, suppression of KU70 and DNA ligase IV by short-hairpin-RNA-based interference increases HDR efficiency by four- to five-fold [16]. Treatment with Scr7, a DNA ligase IV inhibitor, increased the efficiency of HDR-mediated genome editing using Cas9 in mammalian cell lines and in mice. Class 2 CRISPR systems are found in diverse bacterial and archaeal life and endow them with diverse mechanisms for adaptive immunity. Konermann et al. identified a novel family of RNA-targeting class 2 CRISPR systems that designate Type VI CRISPR-Cas13d systems, which contain two HEPN ribonuclease motifs and exhibited RNA-specific, guide-sequence dependent cleavage activity [17]. Then CasRx (*Ruminococcus flavefaciens* Cas13d), which were engineered from optimized versions of several Cas13d orthologs for expression in human cells, showed the maximum efficiency in cleaving target sequences. CasRx exhibited higher efficiency and specificity compared with traditional short-hairpin-RNA-based interference across diverse endogenous transcripts, which positions CasRx as a programmable RNA-binding module for post-transcriptional gene silencing. We thus hypothesized that inhibition of DNA ligase IV by CasRx should improve the efficiency of HDR. In addition, cyclic GMP–AMP synthase (cGAS) is a cytosolic DNA sensor that activates innate immunity by initiating the STING–interferon regulatory factor (IRF) 3 (IRF3)-type I IFN signaling pathway [18]. Nuclear translocation of cGAS induced by DNA damage is uncoupled from the function of cGAS in DNA sensing. Nuclear cGAS can inhibit HR but not NHEJ without altering the cell cycle or DNA replication [19]. Furthermore, Kaposi’s sarcoma-associated herpesvirus (KSHV) is known to encode multiple genes with immune modulatory functions, and initial screening found at least eight ORFs that interfered with the cGAS-STING signaling pathway. However, unlike ORF52, an abundant γ herpes virus-specific tegument protein which is found exclusively in the cytoplasm and targets cGAS directly and inhibits cGAS-dependent antiviral responses, these KSHV proteins appear to interfere with downstream signaling components of cGAS, including IRFs [20–23]. KSHV ORF52, as an inhibitor of cGAS, can subvert cytosolic DNA sensing by directly inhibiting cGAS enzymatic activity through a mechanism that involves both cGAS binding and DNA binding [24]. Together, these functions provide a strategy in which co-expression of an nuclear localization signal (NLS)-ORF52 fusion with the Cas9 system can improve the efficiency of HDR. We now show that the efficiency of HDR can be promoted by suppression of DNA ligase IV through the CasRx system, or by KSHV ORF52 when co-expressed with the Cas9 system in mouse cell lines.

## Materials and methods

### EGFP light reporter plasmid construct

The reporter expression construct was assembled by cloning PCR fragments including the CAG promoter, EGFP gene linked to the 2A peptide from *Thosea asigna*, and the puromycin gene cloned between the 800 bp-5′ *Rosa26* homologous arm and 800 bp-3′ *Rosa26* homologous arm. The donor DNA required for HDR-based repair of this reporter was supplied with the SSODN with a 60-bp homologous arm sequence that was identical with EGFP except for C>G and T>C transitions, corresponding to two consecutive amino acid changes (T67S and Y68H). The sgRNA target along with EGFP were cloned by oligonucleotide annealing as previously reported [25].

### Generation of CRISPR-Cas9 vector expressing ORF52 proteins and NLS-optimized CasRx

To obtain CRISPR-Cas9-T2A-mCherry-P2A-NLS-ORF52 co-expression plasmids, the coding regions for ORF52 proteins were synthesized as mammalian codon-optimized sequences by Sangon Biotech (Shanghai). The ORF52 sequences are listed in Supplementary Table S4. Using ORF52 genes and mCherry template plasmids, we amplified T2A-mCherry-P2A- NLS-ORF52 fragments and cloned them into the pX330 (Addgene, cat. no. 42230) plasmid by Gibson assembly (New England Biolabs, E2611S). To generate NLS-optimized CasRx vectors, we fused CasRx to different NLSs and cloned it into the CAG-sv40NLS-Cas13d-sv40NLS-p2a-GFP plasmid.

### sgRNA and pre-gRNAs

sgRNAs were designed based on unique sequences with 20 nt and complementary oligonucleotides were ordered separately, annealed and cloned into the BbsI sites of the CRISPR-Cas9-T2A reporter plasmid. Pre-gRNAs were designed based on unique sequences with 20 nt and complementary oligonucleotides were ordered separately, annealed and cloned into the BbsI sites of the CasRx pre-gRNA cloning backbone. The sequences used in the present study for gene targeting are listed in Supplementary Tables S1 and S2.

### Cell culture and reagents

Wild-type N2A cells and N2A Rosa26^EGFP+^ cells were maintained in Dulbecco’s modified Eagle’s medium (DMEM) (Gibco) supplied with 10% fetal bovine serum (FBS) (Gibco) and passaged three times per week. The mouse E14 cells were maintained in 2i medium, comprising DMEM (Gibco, 11965-092) containing 15% FBS (Gibco), 1000 U/ml mouse Lif, 2 mM glutamine (Sigma), 1% penicillin/streptomycin (Thermo Fisher Scientific), 0.1 mM β-mercaptoethanol (Sigma), 0.1 mM non-essential amino acids (Gibco), 1 μM PD0325901 and 3 μM CHIR99021. For puromycin selection, EGFP+ cells were sorted, seeded at 10^3^ cells/well and selected with 2 mg/ml of puromycin for more than 2 weeks (Supplementary Figure S1A).

### Donor vectors

The lengths of homologous arms for the donor constructs were set at approximately 800 bp. The donor constructs for the genome editing of ACTB, Nanong and SOX2 loci were generated by overlapping PCR with the genomic DNA of mouse N2A cells as the template.

### Transient transfection

For reporter assays, N2A cells were transfected in a 24-well format with 500 ng of CasRx expression plasmid, 500 ng of guide expression plasmid and 60 ng of mCherry expression plasmid with Lipofectamine 3000 (Life Technologies). Cells were harvested after 72 h and analyzed by flow cytometry.

For transient transfection, N2A cells were plated in a 24-well plate and transfected at >90% confluence with 500 ng of CasRx expression plasmid and 500 ng of gRNA expression plasmid using Lipofectamine 3000 (Life Technologies) according to the manufacturer’s protocol. Transfected cells were harvested 72 h post-transfection for flow cytometry, gene expression analysis or other downstream processing.

For EGFP reporter assays, *Rosa26*^EGFP^ N2A cells were seeded in a 12-well format. Cells were transfected using the Lipofectamine 3000 reagent (Life Technologies) following the manufacturer’s protocol with different plasmid groups as shown in the present paper. Two or three parallel transfections were performed for each plasmid group, and for each well approximately 2 μg plasmids were used.

### RNA extraction and real-time quantitative PCR

Total RNA was isolated from the cells using the TRIzol reagent (Invitrogen). The cDNA was obtained from approximately 1 μg RNA and reverse transcribed by the PrimeScript™ RT Reagent Kit with gDNA Eraser (Takara). Real-time quantitative PCRs (rtqPCR) were performed on a Bio-Rad CFX96 using the SYBR Green Mix (TAKARA) in triplicate. All the gene expression levels were normalized to the internal standard gene, *Gapdh*. The primer sequences are listed in Supplementary Table S3.

### Genotyping analysis

Cells were sorted based on fluorescence and transferred directly into PCR tubes containing 20 μl of lysis buffer (0.1% Tween 20, 0.1% Triton X-100 and 4 μg/ml proteinase K). The samples were incubated for 30 min at 56°C and proteinase K heat-inactivated at 95°C for 10 min. PCR amplification was performed using nested primer sets (Supplementary Table S3). ExTaq was activated at 95°C for 3 min, and PCR was performed for 30 cycles at 95°C for 15 s, 58°C for 15 s and 72°C for 1 min, with a final extension at 72°C for 2 min. Secondary PCR was performed using 1 μl of primary PCR product and a nested inner primer. PCR was carried out in the same reaction mixture. The PCR product was gel purified and sequenced.

### Identification of off-target sites

Putative off-target sites were identified using the CRISPR Design Tool (http://crispr.mit.edu) and the BLAST algorithm against the mouse genome. The identified off-target loci were amplified with the primers listed in Supplementary Tables S5 and S6. Sanger sequencing was applied to the PCR products to confirm the presence of double peaks.

### Statistical analysis

All statistical values are presented as mean ± SEM. Differences between datasets were judged to be significant at: *P<0.05, **P<0.01, ***P<0.001.

## Results and discussion

**Figure 1:**
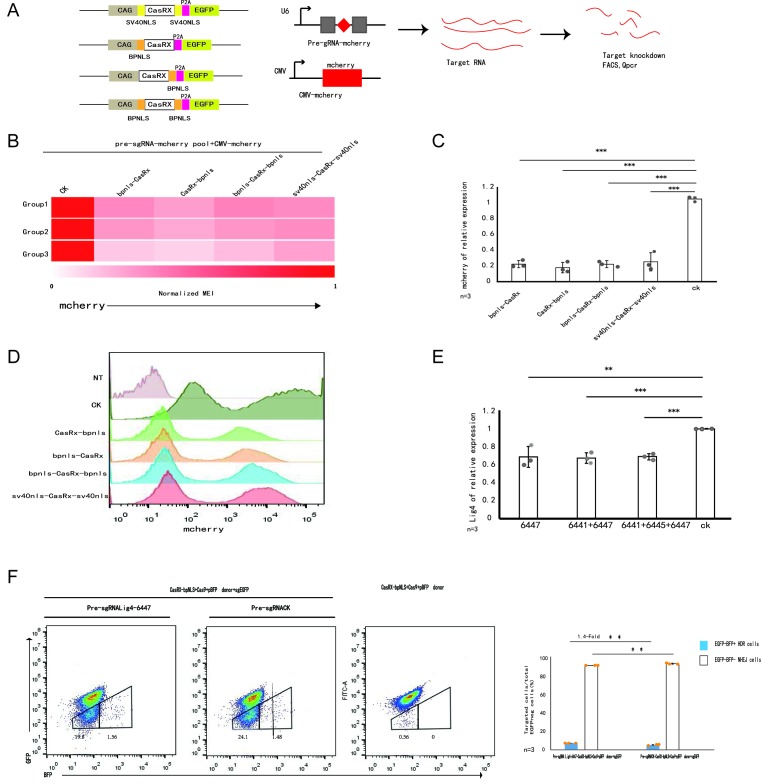
Enhancement of HDR through suppression of ligase IV in the CasRx system for CRISPR-Cas9-induced precise gene targeting (**A**) Schematic for mammalian expression constructs encoding for CasRx effectors and guides. Abbreviation: pre-gRNA, artificial unprocessed guide RNA containing a single 30-nt spacer sequence flanked by two full-length 36-nt DRs. (**B**) Heatmap of mCherry protein knockdown in a CasRx ortholog activity screen in N2A cells using pools of four pre-gRNAs. Normalized MFI, normalized median fluorescent intensity, relative to non-targeting conditions, with *n*=3 (Group1, Group2, Group3). (**C**) Transcription analysis of mCherry genes. The expression values were normalized to that of Gapdh. Data are shown as the average mean ± s.d. (*n*=3 independent experiments). The source data can be found in Supplementary Table S5. (**D**) Fluorescence intensities of mCherry using different engineered CasRx. All four groups showed mCherry knockdown compared with the control group. (**E**) The efficiency of knockdown of endogenous Lig4 of three groups. Group-1 were injected 6447 only, Group-2 were given 6441 and 6447, while Group-3 were injected with 6441, 6447 and 6445. The three groups showed similar efficiencies compared with the control group. The source data can be found in Supplementary Table S5. (**F**) Improvement of HDR efficiency by suppression of NHEJ key molecules. N2A-Rosa26^egfp+^ cells were transfected with CasRx-bpNLS/mCherry, sgGFP/Cas9/mCherry expression vector and the pBFP donor plasmid without or together with coexpression of pre-sgLig4-6447. The frequency of GFP–BFP− (white bars) and GFP–BFP+ (blue bars) cells was analyzed at day 3, with *n*=3. The graph summarizes the results from three independent experiments with similar results. The bars represent mean values ± s.d. Significance was calculated using unpaired Student’s *t* test: *P<0.05, **P<0.01, ***P<0.001, ns, not significant. The source data can be found in Supplementary Table S5.

In fusing Cas9 or BE4 to different NLSs, the bipartite NLS (bpNLS) was superior to SV40NLS in Cas9 or BE4 for nuclear targeting and genome editing [26,27]. First, to further enhance CasRx activity, we tested all five combinations of CasRx N- and C-terminal fusions to the SV40 NLS used in CasRx or to a bpNLS, then assessed the ability of the five CasRx systems to knock down mCherry protein levels in N2A cells using pools of four pre-gRNAs and the CMV-mCherry donor (four pre-gRNAs and the CMV-mCherry donor only as a control) (Figure 1A). At 72 h post-transfection, qPCR analysis indicated that bpNLS-CasRx, CasRx-bpNLS, bpNLS-CasRx-bpNLS, sv40NLS-CasRx-sv40NLS efficiently knocked down mCherry protein levels by up to 78, 82, 78 and 75% (*P*<0.001), respectively (Figure 1C and Supplementary Figure S1A). Flow cytometry also showed that CasRx-bpNLS performed best and resulted in the lowest median fluorescence intensity of mCherry relative to the control group (*n*=3 independent experiments, Group1, Group2 and Group3). (Figure 1B,D and Supplementary Figure S1B). Next, we tested whether CasRx-bpNLS works in mouse cells by using the DNA ligase IV gene, a key enzyme for NHEJ, as a potential target for improving HDR efficiency. Three pre-sgRNAs (6441, 6445, 6447) were tested in N2A cells, and showed highly efficient knockdown of Lig4 protein levels, although pre-sgRNA-lig46447 performed best (Supplementary Figure S1C). CasRx-bpNLS together with pre-sgRNA-lig46447, pre-sgRNA-lig46441+6447 or pre-sgRNA-lig46441+6447+6445 was transfected in N2a cells, and the difference in knockdown DNA ligase IV was compared among the three groups. After 72 h, the qPCR results were similar for each group, and efficiently knocked down LIG4 protein levels by 31, 32 and 31% (*P*<0.01), respectively (Figure 1E). No statistically significant differences were detected between the three experimental groups, thus we chose pre-sgRNA-lig46447 to knock down DNA ligase IV.

**Figure 2:**
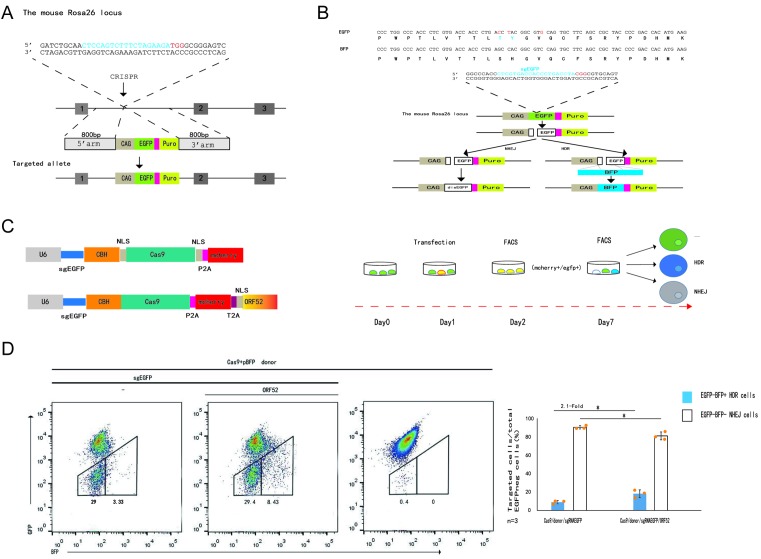
Enhancement of HDR by co-expression of ORF52 for CRISPR-Cas9-induced precise gene targeting (**A**) Strategy for insertion of an EGFP reporter gene into the mouse Rosa26 locus using CRISPR-Cas9 in mouse N2A cells. In the targeted sequence, the Rosa26-specific sgRNA is indicated in blue and the PAM signal is shown in red. In the Rosa26-EGFP targeting vector, the EGFP gene is flanked by Rosa26 homology regions of 800 bp. (**B**) Fluorescent reporter replacement in the mouse N2A cells. The cell line harbors a CAG-GFP cassette in the Rosa26 locus. In the targeted GFP sequence, the sgGFP target sequence is highlighted in blue and the PAM element in red. (**C**) Enhancement of HDR repair using the co-expression of ORF52 proteins. DSBs repaired by the NHEJ pathway led to the inactivation of GFP. DSBs repaired by HDR with the pBFP donor template led to the replacement of GFP by the BFP reporter gene. (**D**) N2A-Rosa26^egfp^ cells were transfected with sgGFP/Cas9/mCherry expression vector and the pBFP donor plasmid without or together with coexpression of ORF52. The frequency of GFP–BFP– (white bars) and GFP–BFP+ (blue bars) cells was analyzed at day 3, with *n*=3. The graph summarizes the results from three independent experiments with similar results. The bars represent mean values ± s.d. Significance was calculated using unpaired Student’s *t* test: *P<0.05; ns, not significant. The source data can be found in Supplementary Table S5.

To measure HDR efficiencies of CRISPR-Cas-induced DSB repair in a reliable and simple manner, we first generated a fluorescent reporter system that was integrated into the *Rosa26* loci in mouse N2A cells (Figure 2A,B and Supplementary Figure S2A). N2A cells were transfected with a *Rosa26* targeting vector carrying the fluorescent reporter system insert, expression plasmids for Cas9, and a *Rosa26*-specific sgRNA (Figure 2A and Supplementary Figure S2A). The reporter included a CAG promoter for expression of a green fluorescent (EGFP) gene and a puromycin gene. For a homologous template, we used a BFP donor plasmid that was identical with EGFP except for C>G and T>C transitions, corresponding to two consecutive amino acid changes (T67S and Y68H). This is sufficient for conversion of EGFP into BFP reporter expression. For activation of the reporter, we designed an sgRNA that specifically recognizes EGFP to induce deletions (Figure 2B). Using this system in mouse N2A cells stably expressing EGFP, we simultaneously estimated both HR (as determined by the number of BFP+ cells) and NHEJ frequencies (as determined by the number of EGFP-/BFP-cells) in the total population of transfected cells (Figure 2C). We designed three groups in N2a Rosa26^EGFP+^ cells to test whether the inhibition of DNA ligase IV by the CosRx system can improve the efficiency of HDR (Figure 1F). After 72 h we analyzed the frequency of BFP+ and EGFP-/BFP-cells within mCherry+ cells by FACS. BFP+ (HDR) cells increased from 1.48% for CasRx-bpNLS/sgCK(control)/Cas9-sgEGFP/BFP to 1.56% in the presence of pre-sgRNA-lig46447 against DNA ligase IV, EGFP-/BFP-cells (NHEJ) decreased from 24% in the presence of CasRx-bpNLS/sgCK(control)/Cas9-sgEGFP/BFP, and further decreased to 19.8% for pre-sgRNA-lig46447/CasRx-bpNLS/Cas9-sgEGFP/BFP (Figure 1F). The other two independent experiments also showed similar results (Supplementary Figure S1D). Statistical analysis results of the three independent experiments demonstrated 7.7% HDR efficiency for the pre-sgRNA-lig46447/CasRx-bpNLS/Cas9-sgEGFP/BFP groups, which amounted to a 1.4-fold expression increase compared with the control groups without pre-sgRNA-lig46447 expression (*P*<0.01), while the frequency of NHEJ editing was 0.9-fold that of the control (*P*<0.01). (Figure 1F). Consistent with our hypothesis, these results show that the CasRx system is effective in enhancing HDR efficiency by suppressing DNA ligase IV.

As mentioned previously [19,24,28], nuclear cGAS can suppress homologous-recombination-mediated repair and KSHV ORF52, as an inhibitor of cGAS, can subvert cytosolic DNA sensing by directly inhibiting cGAS enzymatic activity. Therefore, we next inhibited the nuclear cGAS by KSHV ORF52 to assess the effect of nuclear KSHV ORF52 on improving the efficiency of HDR. We co-transfected N2a Rosa26^EGFP+^ cells with an EGFP-specific sgRNA/Cas9/mCherry (sgEGFP/Cas9/mCherry) expression plasmid and the BFP vector, with or without co-expression of NLS-ORF52 proteins (Figure 2C). After 24 h, we sorted the cells transfected with sgGFP and Cas9 or Cas9-NLS-ORF52, as determined by mCherry. Approximately 38% of the transfected cells lost GFP expression after 72 h, and approximately 8.43% of the GFP-cells were BFP+ for sgEGFP/Cas9-NLS-ORF52/BFP. Approximately 32% of the transfected cells lost GFP expression after 72 h and 3.33% of the GFP-cells were BFP+ for sgEGFP/Cas9-NLS-ORF52/BFP (Figure 2D). The other two independent experiments showed similar results (Supplementary Figure S2B). Statistical analysis results of the three independent experiments demonstrated 9% HDR efficiency for the sgEGFP/Cas9-NLS-ORF52/BFP groups, which corresponded to 2.1-fold of the control groups without NLS-ORF52 expression (*P*<0.05), while the frequency of Cas9-NLS-ORF52-mediated NHEJ editing was 0.9-fold of the control (*P*<0.05) (Figure 2D). Taken together, we concluded that co-expression with NLS-ORF52 could enhance the HDR-based genome editing by more than two-fold. As an additional benefit, the co-expression strategy was potentially capable of inhibiting the NHEJ effect to some extent. Thus, co-expression with NLS-ORF52 can enhance HDR efficiency to a greater extent than using the CasRx system mediated suppression of DNA ligase IV.

**Figure 3:**
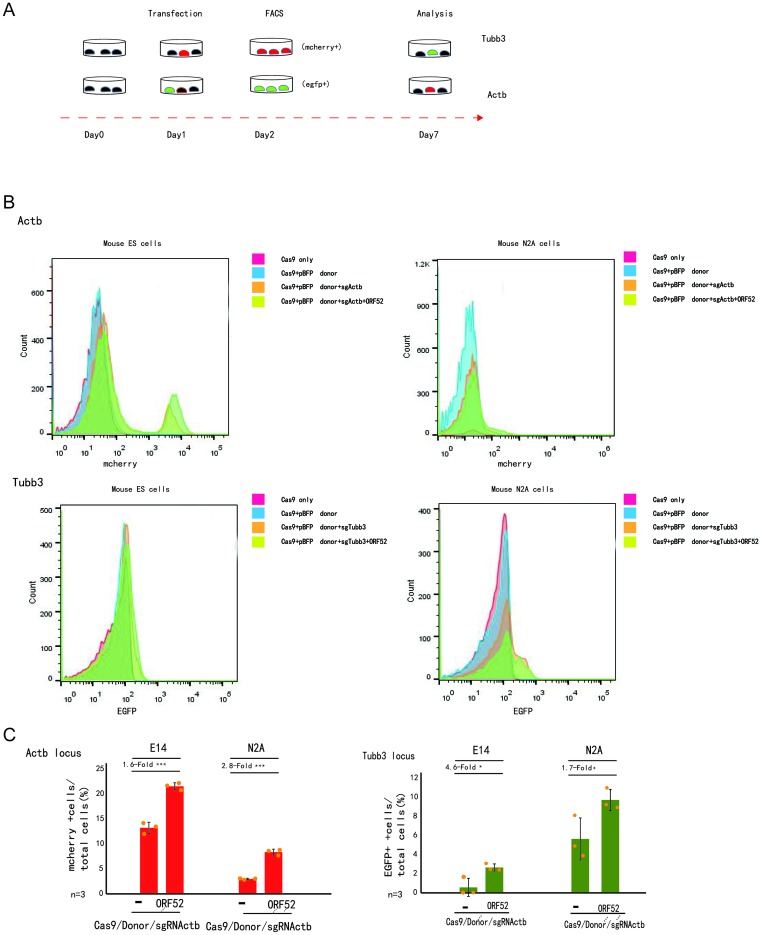
*In vitro* genome editing via ORF52-mediated targeted integration (**A**) Experimental scheme for targeted Actb-2A-mCherry and Tubb3-EGFP knockin in mouse ES cells or N2A cells. Cells were transfected with donors/sgRNA/GFP or donors/sgRNA/mCherry and Cas9 or Cas9/ORF52, and transfected cells were sorted based on GFP or mCherry signals 2 days after transfections. Knockin efficiencies were evaluated by FACS based on the ratio of GFP+ or mCherry+ cells among total transfected cells 4 days after the first sorting. (**B**) Fluorescence intensities of HDR products in mouse ES cells or N2A cells. Co-expression of ORF52 yields an HDR product with greater fluorescence than no ORF52 in Actb or Tubb3 loci (related to Supplementary Figure S2C,D). (**C**) Histograms show relative knockin efficiencies of co-expression of ORF52 or no ORF52-based knockin strategies in mouse cells at Actb and Tubb3 loci measured by the percentage of mCherry+ (or GFP+) cells among total transfected cells, with *n*=3. The bars represent mean values ± s.d. Significance was calculated using unpaired Student’s *t* test: *P<0.05, ***P<0.001; ns, not significant. The source data can be found in Supplementary Table S5.

We examined whether the ORF52 protein showed a robust knockin in endogenous genes by fusing a p2A-mCherry and EGFP reporter gene to the last codon of the *Actb* and *Tubb3* genes together with an HR donor (800 bp arm) in mouse N2a or E14 cells (Figure 3A and Supplementary Figure S3A). The resulting knockin efficiencies are presented as percentages of mCherry+ cells or EGFP+ cells. At 7 days after transfection, our data showed that co-expression with NLS-ORF52 can improve the efficiency of HDR across Actb and Tubb3 loci in mouse ES cells. However, low fluorescence intensity and a low HDR frequency were observed in comparison with the control group without NLS-ORF52 (Figure 3B,C). Mouse ES cells with NLS-ORF52 yield an HDR product of 1.8× or 1.85× stronger fluorescence than the control group in Actb and Tubb3 loci, respectively (Figure 3B and Supplementary Figure S2C). The other two independent experiments provided similar results (Supplementary Figure S2C). Similar results were obtained in mouse N2A cells, mouse N2A cells with donor/sgRNA plasmids, and Cas9-ORF52 (Figure 3B,C). Mouse N2A cells with NLS-ORF52 yield an HDR product of 2.8× or 1.35× stronger fluorescence than the control group without NLS-ORF52 in Actb and Tubb3 loci, respectively (Figure 3B and Supplementary Figure S2D). The other two independent experiments provided similar results (Supplementary Figure S2D). Statistical analysis demonstrated, whether in N2A cells or in E14 cells, that co-expression with NLS-ORF52 could significantly enhance the HDR efficiency compared with the control groups without ORF52 expression in Actb and Tubb3 loci. The ORF52 co-expression strategy yielded 21.7 and 2.7% HDR efficiency at the Actb and Tubb3 loci, respectively, which corresponded to 1.6-fold (*P*<0.001) and 4.6-fold (*P*<0.05) of the control groups in mouse E14 cells. Alternatively, the ORF52 co-expression strategy exhibited good performance with HDR efficiencies of 8.3 and 9.7%, corresponding to 2.8-fold (*P*<0.001) and 1.7-fold (*P*<0.05) of the control groups, respectively, at the Actb and Tubb3 loci in mouse N2A cells (Figure 3C). Genotyping showed that Actb- or Tubb3-mediated gene knockin represents precise in-frame integration at the 5′ and 3′ junctions (Supplementary Figure S3B). Thus, the ORF52 protein also can improve HDR efficiency in endogenous genes.

Finally, to assess off-target effects, we performed Sanger sequencing to identify off-target mutations in the Actb and Tubb3 knockin in E14 cell lines. The potential off-target sites of *Actb* and *Tubb3* genes were identified using the CRISPR Design Tool (http://crispr.mit.edu) and the BLAST algorithm, all of which possessed two to three mismatches to the original sgRNA sequences (Supplementary Tables S5 and S6). We observed no off-target effects in the Actb and Tubb3 knockin in E14 cell lines (Supplementary Figure S4).

In summary, the human code optimized CasRx system has been successfully used to knock down mRNA transcripts in human cells, with a higher efficiency and specificity than RNA interference. The same study also demonstrated the therapeutic relevance of RNA knockdown for frontotemporal dementia with Parkinsonism using the CasRx system. Jillette et al. [29] engineered inducible artificial splicing factors (iCASFx) based on CRISPR/dCasRx in human 293T cells. Although there are currently few reports on usage of the CasRx system, it is a promising alternative tool for post-transcriptional gene silencing and shows no detectable side effects in previous research, including toxicity, cell growth or cell death. In our study, CasRx also shows no detectable side effects, including toxicity, cell growth or low efficiency in mouse N2A cells. However, our data, based on only one gene and one type of mouse cell, are insufficient to predict the efficiency and unwanted side effects of the CasRx system generally, and this work must be extended to more mouse genes and cell lines in future studies. In fact, in the present study we also tried CasRx interference in chicken PGCs and selected OCT4 and CVH genes, but unfortunately, slight toxicity in cells led to no detectable stable knockdowns (data not shown). Therefore, whether the human code optimized CasRx system performs high efficiency knockdown with no side effects for other animal cells is a problem that merits further study. In our study we have shown that the suppression of ligase IV with CasRx for improving HDR is not a sufficiently effective route for the genome of mouse cells. As we know, PKcs, Ku70, Ku80, Lig4 and XRCC4 are all key NHEJ factors [30]. Suppression of one key NHEJ factor (besides Lig4) or suppression of multiple key NHEJ factors may be a choice to further improve HDR efficiency. In our investigation into an alternative way to improve the efficiency of HDR, we selected ORF52 proteins of KSHV, shown to specifically inhibit cGAS. Significantly, ORF52 homologs also bound to cGAS and DNA and inhibited cGAS activity directly, suggesting that inhibition of cGAS by virion-contained ORF52 is a conserved mechanism for γ herpesviruses. Using ORF52 we found no detectable side effects, including toxicity, cell growth or cell death in mouse cells in the present study. Although ORF52-mediated HDR efficiency performed better than CasRx-mediated HDR efficiency, this system is not efficient enough. Optimal inhibition of cGAS activity requires both the cGAS-binding and cytosolic DNA-binding properties of ORF52 and the two properties are seemingly inseparable, and the extent to which they contribute to the inhibition of cGAS is unknown. Thus, blocking nuclear cGAS activity, in part by nuclear translocation of the ORF52 NLS fusion constructs, may be the reason for low ORF52-mediated HDR efficiency. Nevertheless, we cannot exclude the possibility that HDR stimulation by the ORF52 proteins is mediated by other regulatory proteins. It could be of further interest to compare the effects of the ORF52 with NLS fusion and ORF52 without NLS fusion on HDR, and to determine whether ORF52 without NLS fusion constructs can inhibit the nuclear translocation of cGAS induced by DSBs. Furthermore, the enhancement effects of the ORF52-Cas9 fusion on HDR will be compared with ORF52 co-expression in future research, and HDR efficiency of the two strategies mediated by different donor templates (plasmid, PCR, and ssDNA) may be possible to further enhance gene targeting efficiency. It will also be interesting to apply CRISPR-Cas9 mutagenesis combined with ORF52 to mouse early embryos to achieve gene corrections. To determine if ORF52 could be safe for enhancing HDR efficiency in mouse embryos, we first need to test the impact of treating embryos with different concentrations. In fact, we had treated mouse embryos with ORF52. Zygotes co-injected with CRISPR-Cas9 components (sgACTB RNA, Cas9 mRNA and PCR targeting template with 800 bp HAs) and 100 ng/μl ORF52 mRNA developed normally to the blastocyst stage with a ∼70% survival rate, comparable with the conditions without ORF52. In addition, we did not see any adverse effects on embryo development. But unfortunately, none of HDR-based precise mutations except the NHEJ-based indels were detected by sequencing (data not shown). However, applying the other donor templates (plasmid and ssDNA) may be an option. Given that, our work still demonstrated the potential of this method for precise genome editing.

## Supplementary Material

Supplementary Figures S1-S4

Supplementary Tables S1-S6

## Abbreviations

bpNLS: bipartite NLS
CasRx: *Ruminococcus flavefaciens* Cas13d
cGAS: cyclic GMP–AMP synthase
DMEM: Dulbecco’s modified Eagle’s medium
DSB: double-strand break
FBS: fetal bovine serum
HDR: homology-directed repair
HEPN: higer eukaryotes and prokaryotes nucleotide-binding
HR: homologous recombination
indel: insertion or deletion
IRF: interferon regulatory factor
KSHV: Kaposi’s sarcoma-associated herpesvirus
NHEJ: non-homologous end-joining
NLS: nuclear localization signal
SSODN: single-stranded oligodeoxynudeotide
