# Supplementary material for: Homology-directed repair in mouse cells increased by CasRx-mediated knockdown or co-expressing Kaposi’s sarcoma-associated herpesvirus ORF52
Source: Biosci Rep. 2019 Oct 15;39(10):BSR20191914. doi: 10.1042/BSR20191914 (PMC6822532; doi:10.1042/BSR20191914)
Supplement: Supplementary Figures S1-S4 [file BSR-2019-1914_supp.pdf]

Supplementary Figure 1.

a

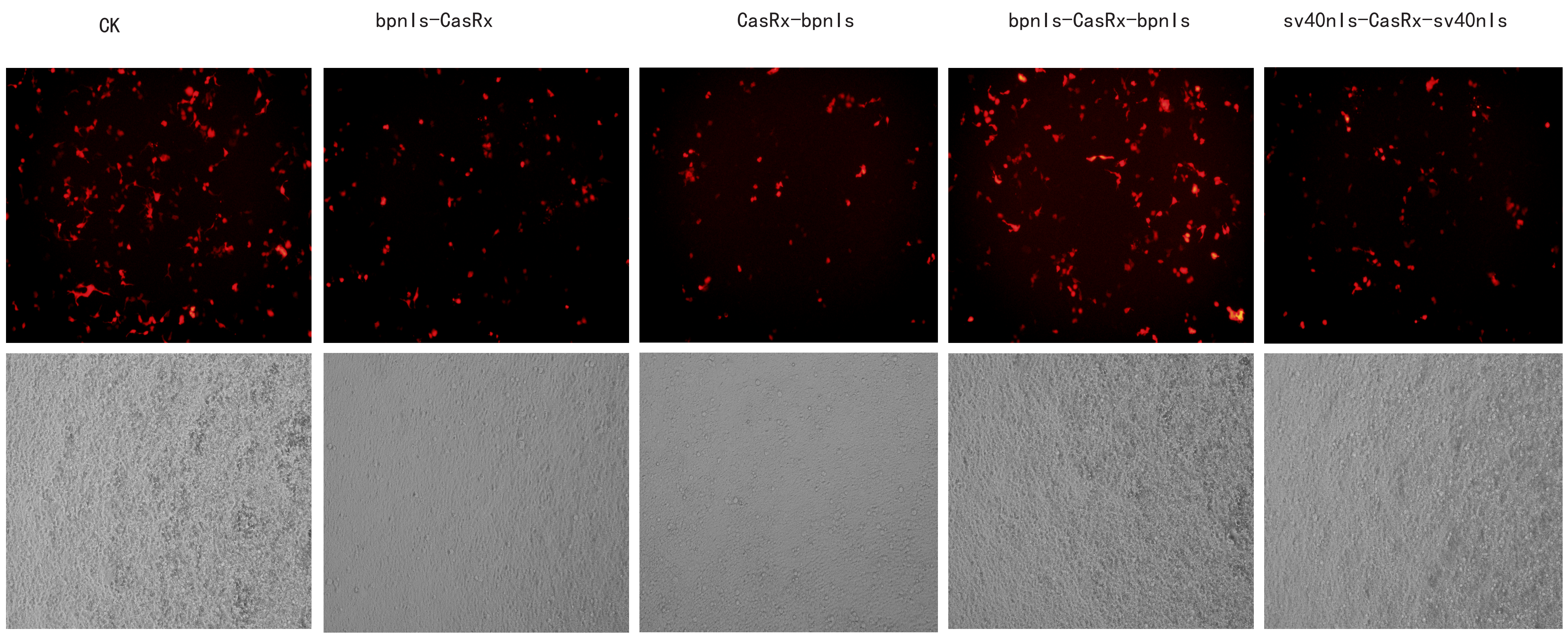

b

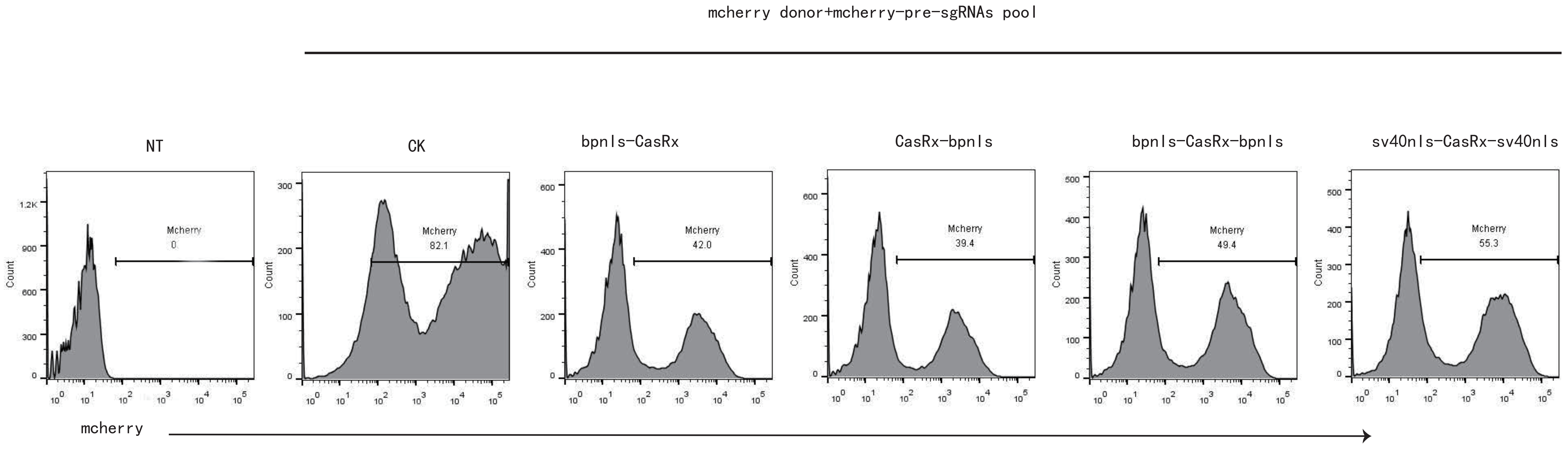

c

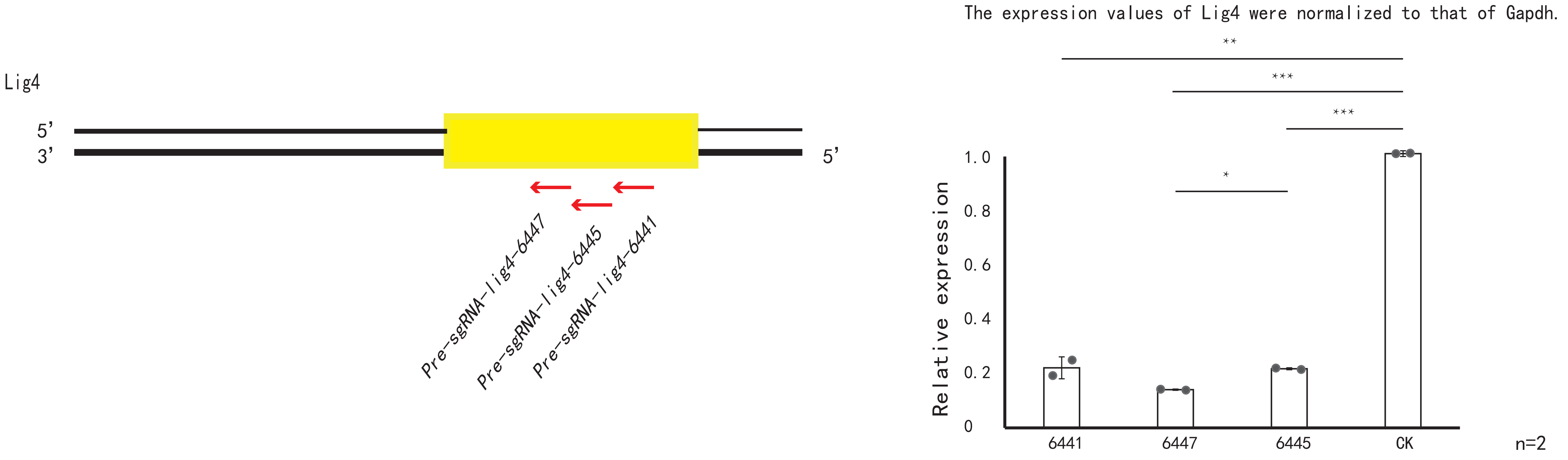

d

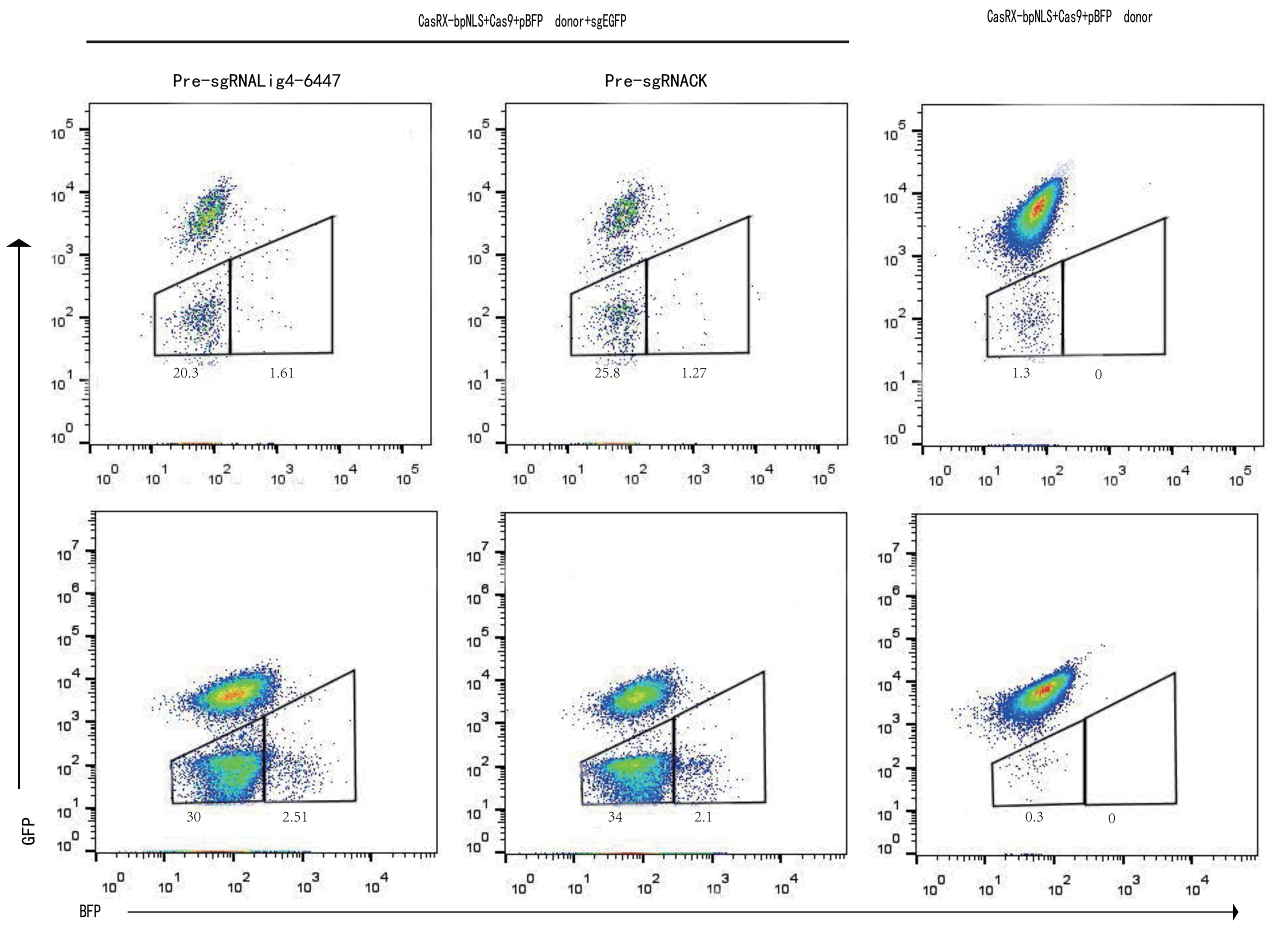

Supplementary Figure 2

a

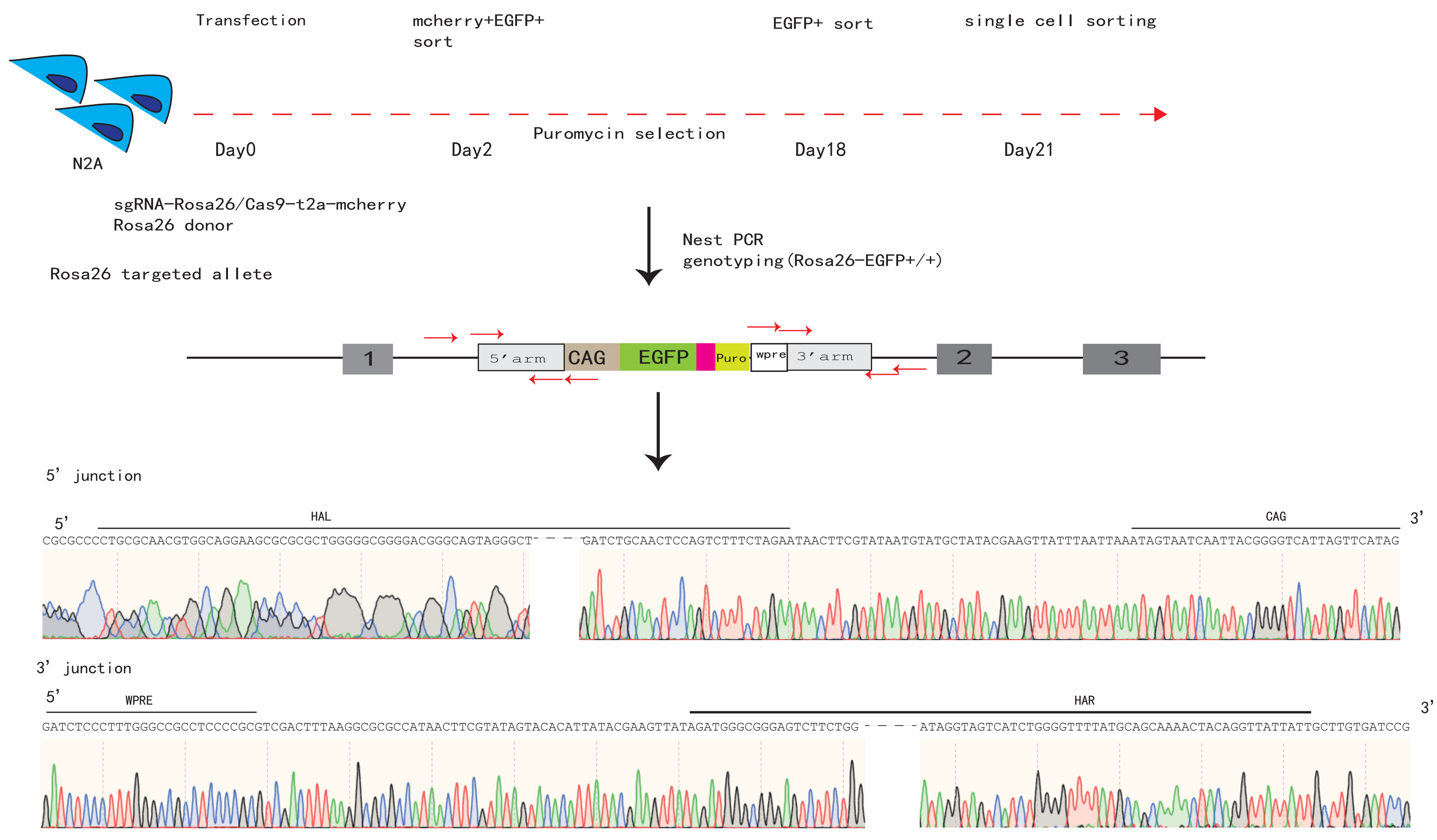

b

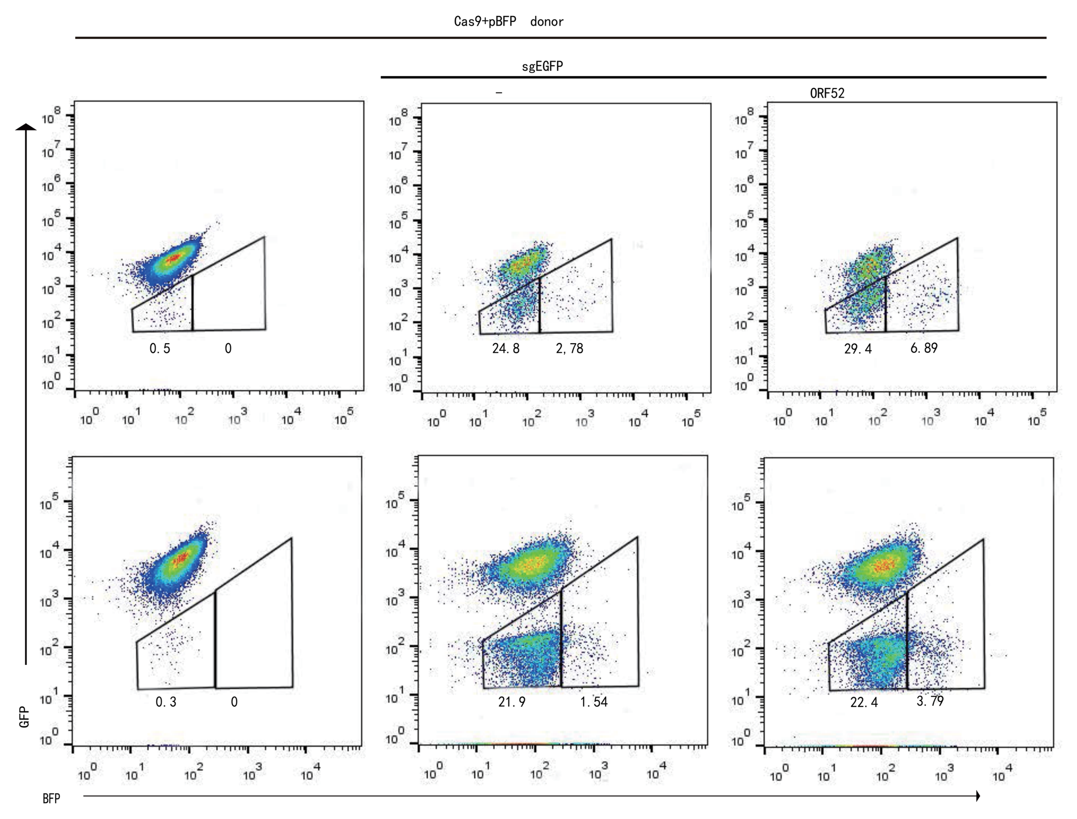

c

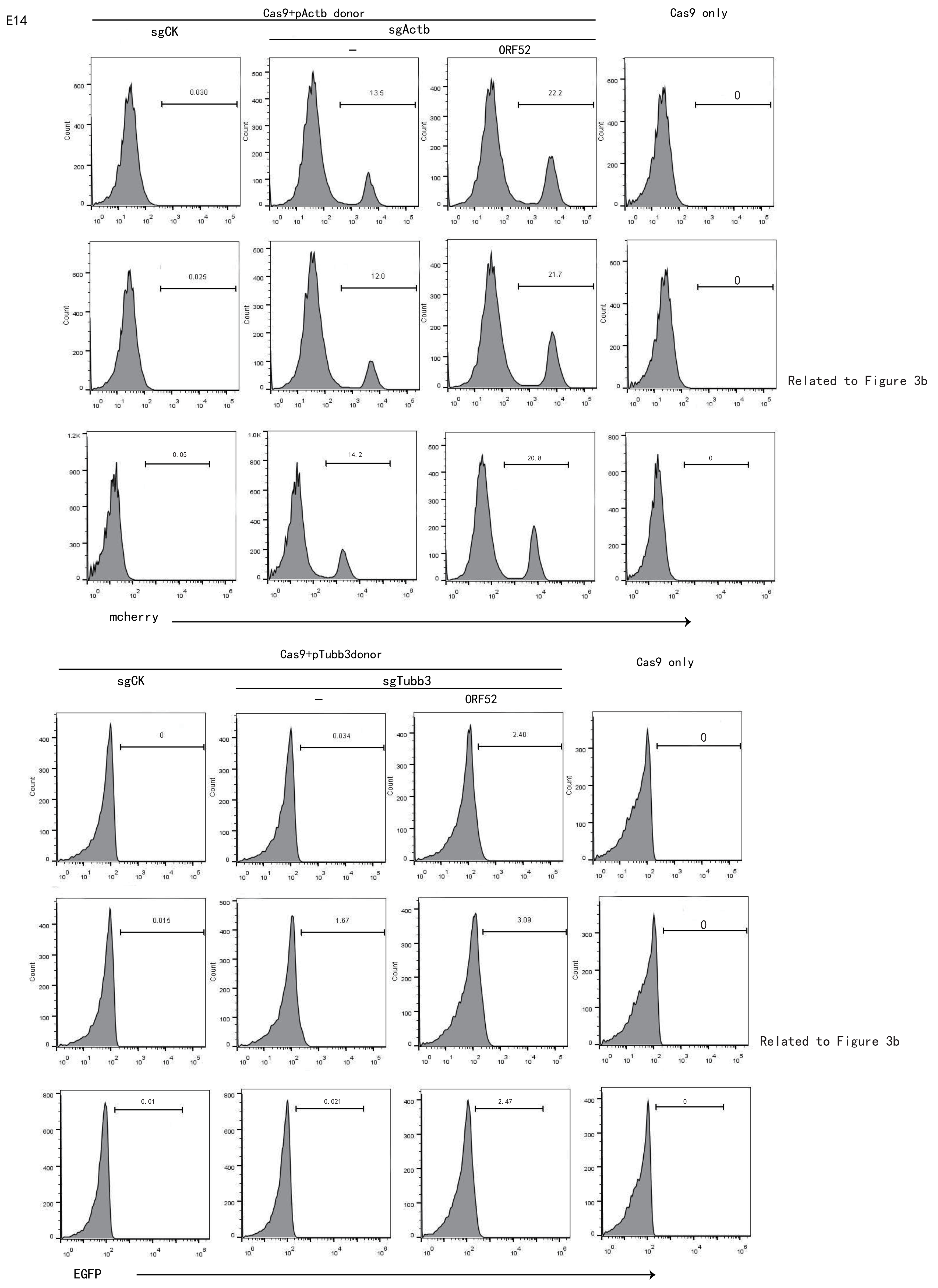

d

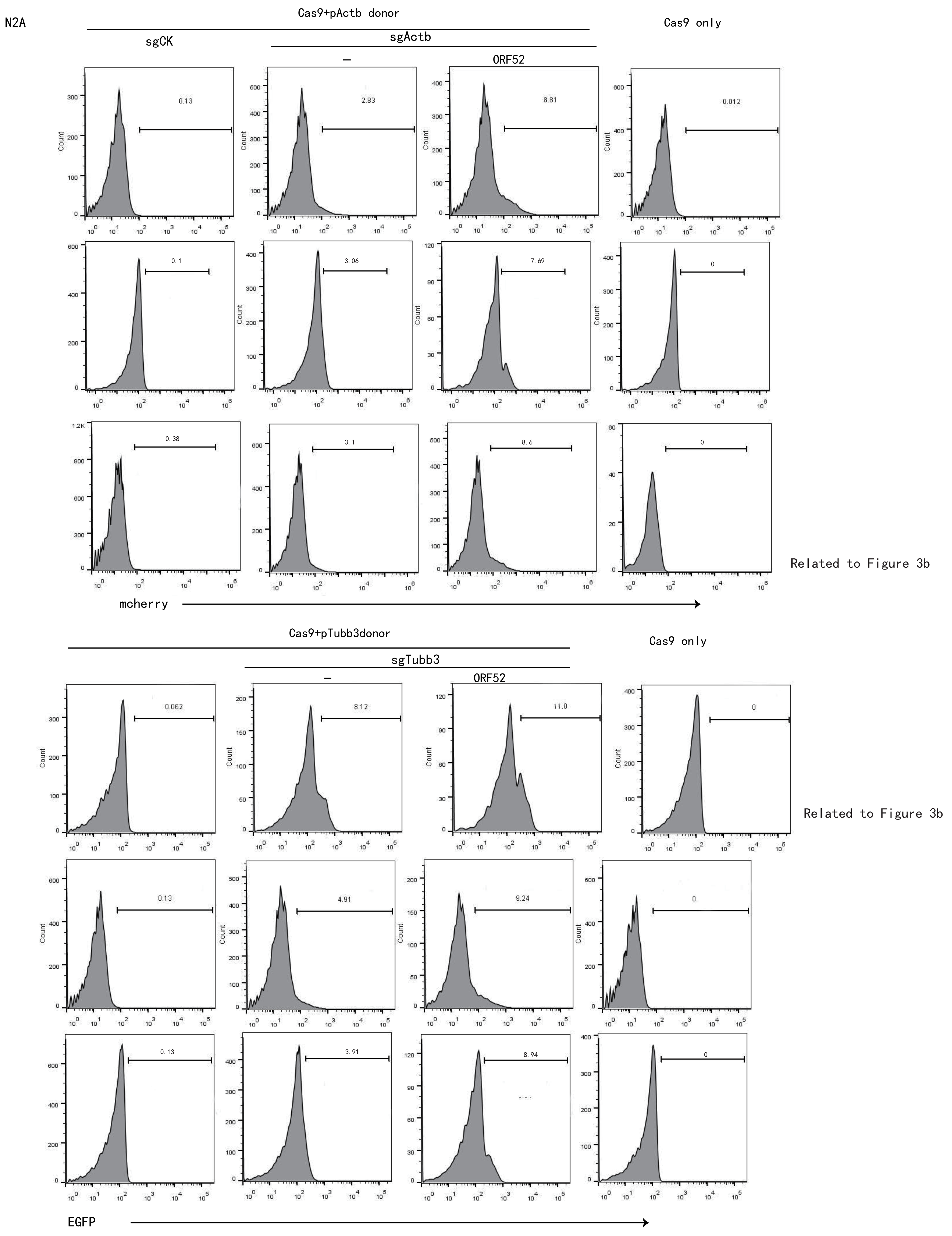

Supplementary Figure 3

a

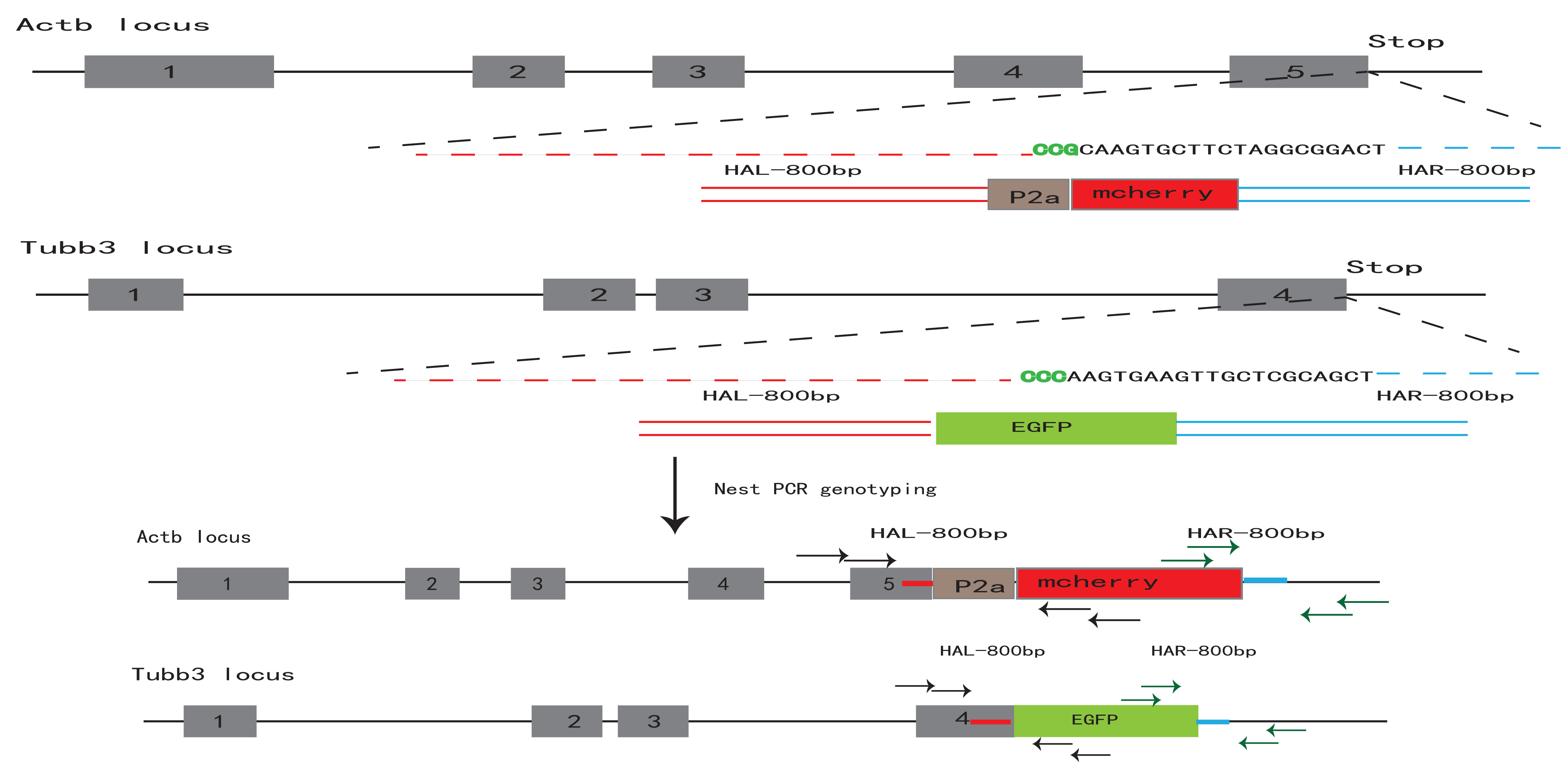

b

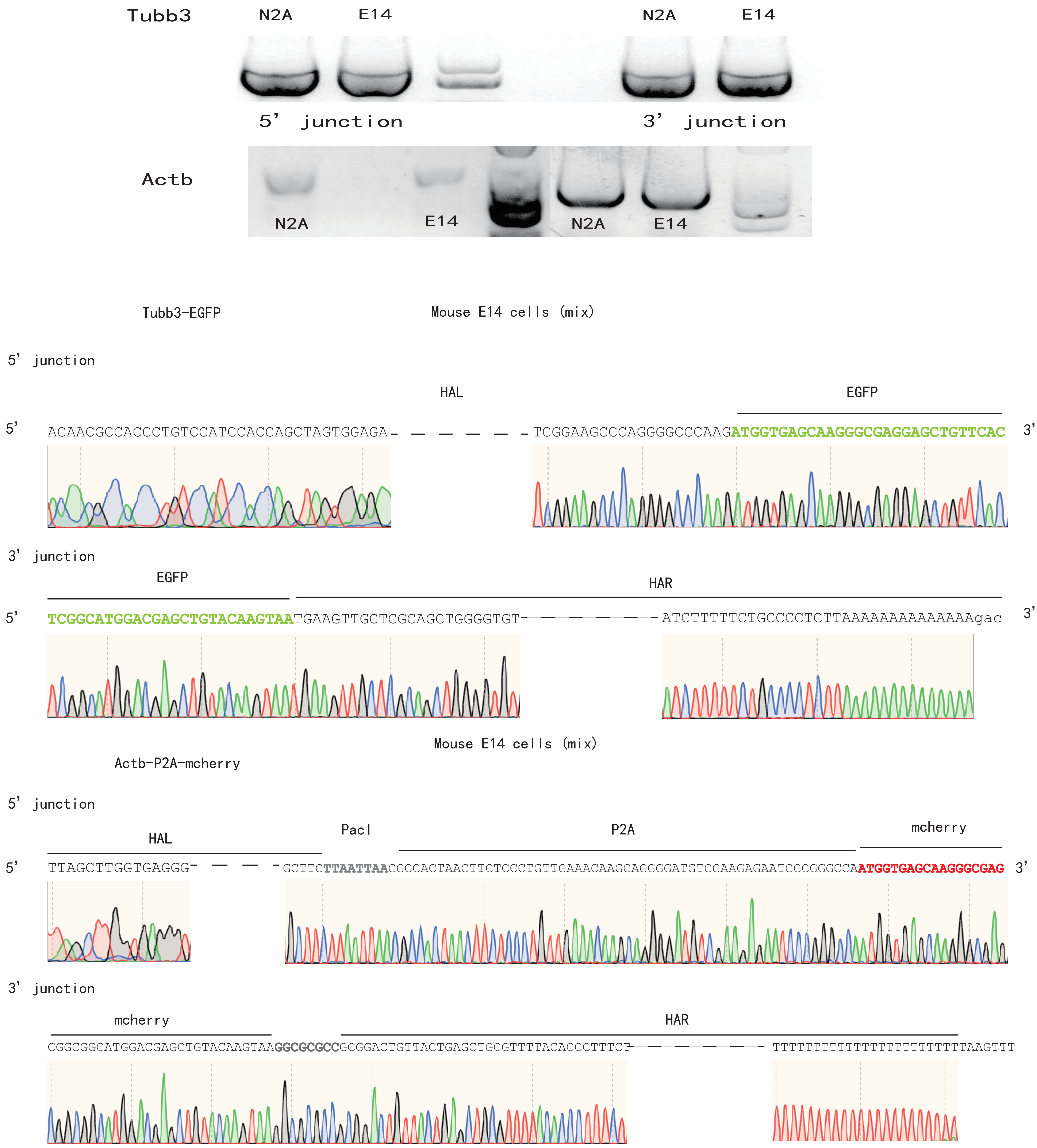

Supplementary Figure 4

ACTB Off-Target

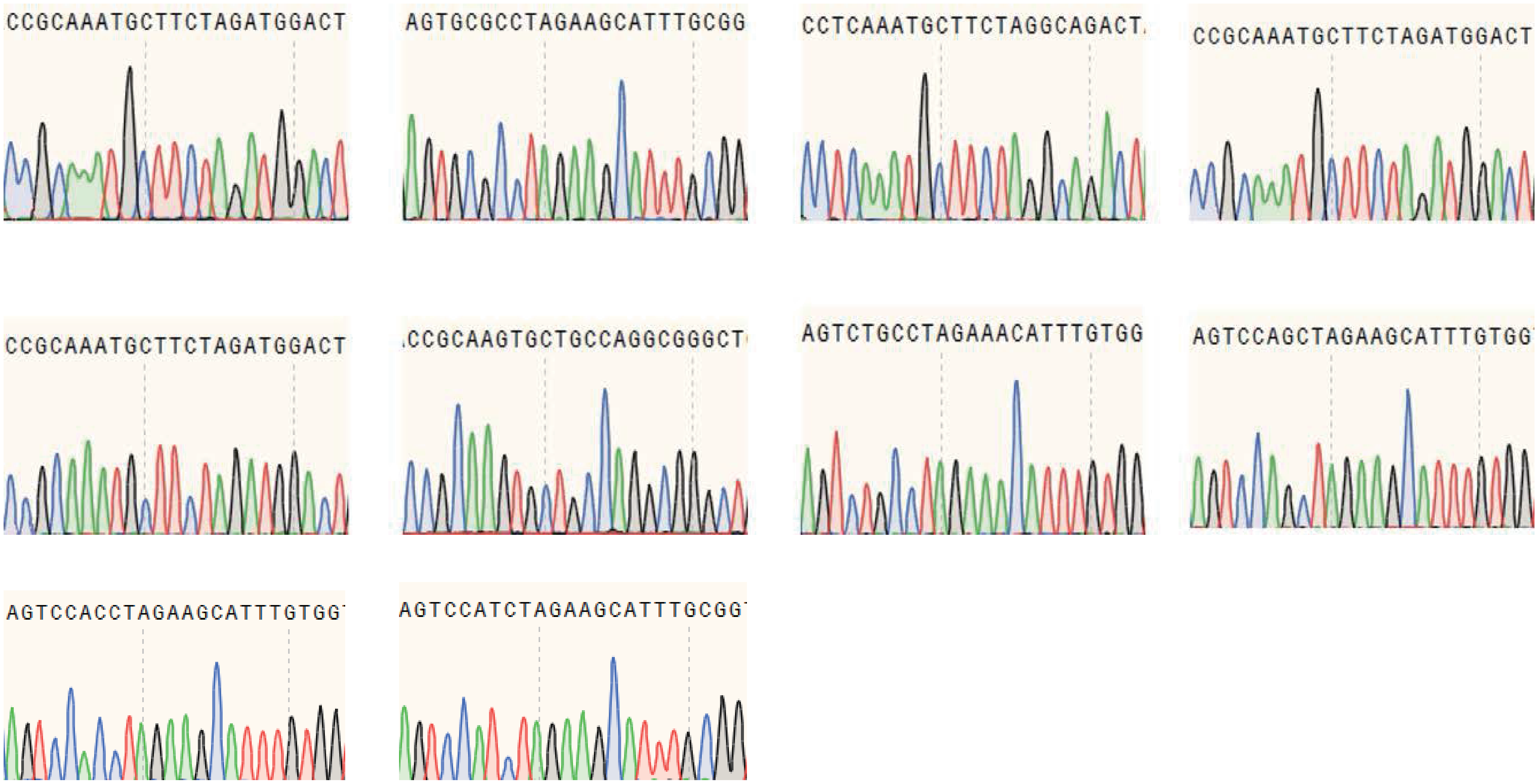

Tubb3 Off-Target

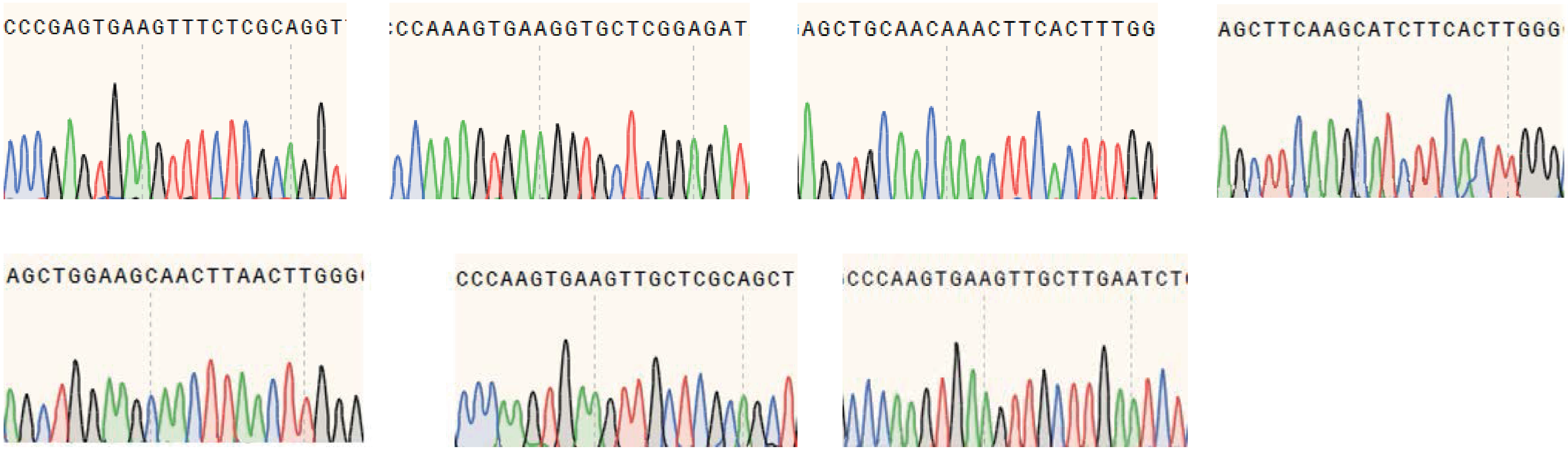

Figure S1 (a) Representative visualization of the mCherry positive cells by fluorescent microscopy. HEK293T cells were co-transfected with different plasmid groups (bpNLS-CasRX, CasRX- bpNLS, bpNLS- CasRX- bpNLS, Sv40NLS- CasRX- Sv40NLS) for knockdown mCherry. The cells were observed and imaged 72h after transfection. Scale bar, 100 µm. (b) One representative of three results of the flow cytometric counting analysis for fluorescent positive cells (related to Figure 1b). (c) Schematic overview of Lig4 RNA targeting strategies. Red Arrow, targeting loci. Comparison of three pre-gRNA-Lig4 (6441,6445 6447) for knockdown of endogenous Lig4 mRNA reveals three pre-gRNA-Lig4 to be similarly effective to CasRX-bpNLS. n=2.The source data can be found in Supplementary Table 5. The bars represent mean values ± s.d. Significance was calculated using the Student's t-test: **P < 0.01, ***P < 0.001, ****P < 0.0001, ns, not significant. (d) Two representative of three results of the flow cytometric counting analysis for fluorescent positive cells (related to Figure 1f).

Figure S2 (a) Strategy for the insertion of the EGFP reporter construct into the Rosa26 locus of N2A cells using CRISPR/Cas9. Genotyping analysis of the cells targeted integration at Rosa26 loci. Red Arrow, primers. PCR products amplified from 5' and 3' junction sites were sequenced. HAL/HAR, left/right homology arm. (b) One representative of two results of the flow cytometric counting analysis for fluorescent positive cells (related to Figure 2d). (c),(d) Two representative of three results of the flow cytometric counting analysis for fluorescent positive cells (related to Figure 3b).

Figure S3 (a)Schematic overview of gene targeting strategies at the Actb and Tubb3 locus. HAL/HAR, left/right homology arm ;Black Arrow, outer forward/reverse primer; Green Arrow, inner forward/reverse primer. (b) Genotyping analysis of cells with coexpression of ORF52 or no ORF52-based targeted integration at Actb and Tubb3 loci in mouse ES or N2A cells. PCR products amplified from 5' and 3' junction sites were sequenced. Upper: homology arm; red, mCherry; green, GFP.

Figure S4. Sanger sequencing chromatograms of off-target sites for Actb and Tubb3 knock-in E14 cell lines.
